# Cutting Through the Clutter: Cleaning the Lens on Work-Related Wellbeing Concepts

**DOI:** 10.3390/bs16030373

**Published:** 2026-03-05

**Authors:** Rona Hart, Dan Hart

**Affiliations:** 1 School of Psychology, University of Sussex, Falmer, Brighton BN1 9RH, UK; 2 Birmingham Business School, University of Birmingham, 116 Edgbaston Park Road, Birmingham B15 2TY, UK; d.hart@bham.ac.uk

**Keywords:** worker wellbeing, work wellbeing, wellbeing at work, employee wellbeing

## Abstract

Work-related wellbeing research is increasingly constrained by conceptual and terminological clutter. Labels such as *worker wellbeing*, *employee wellness*, *wellbeing at work*, *occupational health*, *quality of working life*, and other terms are often treated as synonyms, defined at different levels of abstraction, or operationalised through proxies, limiting cumulative theory and cross-study comparability. This conceptual, semantically-focused, critical paper demonstrates the ways in which legacy terminology, interdisciplinary language gaps, issues with the overarching concept of wellbeing and category errors contributed to the current conceptual and definitional disarray. It then proposes a conceptual map to help resolve this state of affairs. The framework distinguishes overall wellbeing from work-related wellbeing, separates life-domain labels from wellbeing components, clarifies population segmentations, and offers unified set of definitions to these wellbeing constructs. This conceptual work is intended to improve construct selection, specification, and measurement in both research and applied settings. This paper concludes with a future agenda.

## 1. Introduction

The Greek physician Galen asserted that employment is “nature’s physician”, essential to human happiness (Strauss, 1968), hence recognising that purposeful labour is a fundamental element of human physical and psychological flourishing. However, in today’s complex and demanding work environments, such a view may be contested. While employment is vital for mitigating the adverse effects of unemployment and economic hardship, work may not always promote wellbeing or happiness, and can undermine and even actively impair workers’ physical and psychological health. Hence, work can be seen as a double-edged sword, which has the capacity to both enhance and erode workers’ wellbeing.

Given its potential to yield contrasting outcomes for employees, and that these outcomes in turn impinge on organisations’ functioning and bottom lines, it may not be surprising that work-related wellbeing scholarship has spanned a century, and shows no sign of waning.

During this period, research on work/er wellbeing has evolved from an early emphasis on industrial safety and the prevention of occupational disease, through remedial and clinical psychological perspectives, to contemporary humanistic and holistic approaches (Martela, 2025; Wallace, 2022; Hamling et al., 2015), as it shifted from scholarship rooted primarily in management to a broad interdisciplinary field, with psychological perspectives, particularly those drawing on positive work and organisations, currently taking the lead (Martela, 2025).

Work/er wellbeing is increasingly recognised as a central driver of individual functioning, spanning performance, creativity and innovation, productivity, and organisational commitment, and resulting at the organisational level in profitability and sustainability, hence strengthening the business case for continued investment in employee wellbeing (Pandey et al., 2025; Martela, 2025).

Despite the importance and longevity of the concept, recent upsurge in the volume of research, and widespread organisational interest and applications, it has faced repeated critique, with researchers describing the field as “messy” (Wijngaards et al., 2022) and a “conceptual jungle” (Makikangas et al., 2016, p. 62). Fisher (2010) claimed that research in this area is conducted in a manner similar to the proverbial elephant examined by blind men, whereby researchers “have developed a good if isolated understanding of its parts” but they may have “decomposed the beast into almost meaninglessly small pieces” (p. 391). Persistent issues such as multiple incompatible terms, terminological ambiguity, overlapping constructs, multiple and inconsistent definitions or lack of definitions, the absence of a unifying meta-theory, incompatible measures, and the use of numerous incongruent proxies, impede the progression of the field and further innovation, undermining the effective translation of its findings into practice, hence widening the gap between research and practice (Pandey et al., 2025; Wijngaards et al., 2022; Martela, 2025; Pincus, 2024).

In this conceptual, semantically-focused, critical paper we aim to address a key point of critique noted by scholars, but not fully addressed: the multiple concepts and terms used by scholars to refer to work/er wellbeing (for example: *worker wellbeing*, *employee wellbeing*, *occupational wellbeing*, *work wellbeing*, *wellbeing at work*, *work wellness*, *quality of working life*, *employee health*, etc.). Specifically, we summarise and explore the myriad concepts referring to work/er wellbeing, review their definitions, examine the roots or sources of indistinction, and propose a way to address the critique by mapping key terms, and demonstrating their points of convergence and divergence. Through this conceptual mapping exercise we aim to clear the terminological jumble, and clarify, reconfigure and refine this scholarly terrain.

We note that this paper does not offer a complete and exhaustive review or a systematic review of all the concepts that refer to wellbeing in the work sphere. Rather, it focuses on key terms and critical areas of ambiguity that have the potential to advance the field when resolved. Importantly, this paper adopts a predominantly Western lens on work/er wellbeing, as this is the perspective most commonly represented in the literature and where the terminological confusion we seek to address arises.

This paper begins by depicting key concepts that scholars often use to refer to work or worker wellbeing, alongside their definitions, highlighting some areas of vagueness, and reviewing the critiques articulated in the literature on the concepts and their definitions. We then consider the underlying causes or origins of the reported issues. In response to the critique, we propose a conceptual map that demarcates the relationships among these terms, hence distinguishing between distinctive terms and synonymous terms. Finally, this paper highlights current research gaps and outlines directions for future research.

## 2. Work/er Wellbeing: Terminological Clutter

A persistent weakness and a major source of confusion in the work/er wellbeing literature is the abundance of labels for the same underlying ideas. Disaggregating the phrase “work(er) wellbeing” reveals that multiple constructs are used interchangeably with “*wellbeing*” (or well-being in US spelling), *including quality of life, health, welfare, wholebeing and wellness*. Likewise, the “work” referent is variously phrased as *work*, *workplace*, *worker*, *employee*, *personnel*, *staff*, *organisational* or *occupational*. These building blocks are then recombined into a wide array of terms that commonly feature in the literature, including for example *worker wellbeing* (Wijngaards et al., 2022), *employee wellbeing* (Valtonen et al., 2025), *occupational wellbeing* (Saraswati et al., 2019), *work wellbeing* (Jarden et al., 2023), *workplace wellbeing* (Kinowska & Sienkiewicz, 2023), *wellbeing at work* (Clifton & Harter, 2021), *work wellness* (Klerk, 2005), *employee welfare* (Lintong & Bukidz, 2024), *quality of working life* (Horst et al., 2014), *quality of life at work* (Schmidt et al., 2008), *staff wellbeing* (Jayman et al., 2022), *organisational wellbeing* (Kenttä & Virtaharju, 2023), *employee health* (Kleine et al., 2023), and *employee wholebeing* (Rani et al., 2024) to name a few.

Some of these terms are used synonymously in the literature, while others are seen as distinct concepts. For example, in reference to *work wellbeing*, De-Neve and Ward (2025) used the term synonymously with *workplace wellbeing*. Laine and Rinne (2015) conducted a review on *wellbeing at work*, using the term interchangeably with *work wellbeing*. To conduct their review, they used the search terms *wellbeing at work*, *work wellbeing*, *occupational wellbeing* or *employee wellbeing.* Similarly, in their systematic review of measures of *worker wellbeing*, Jarden et al. (2023) used the search terms *worker wellbeing*, *employee wellbeing*, *staff wellbeing* and *personnel wellbeing*.

Daniels et al. (2019) noted that the distinction between *workplace health* and *wellbeing* is fuzzy. Making a similar point Danna and Griffin (1999) observed that researchers often use the terms *psychological wellbeing* and *mental health*, or *physical wellbeing* and *physical health* interchangeably, and argued that the terms employee health and employee wellbeing should not be conflated.

An additional muddle arises from failing to differentiate between context-free constructs, such as *overall wellbeing*, which aggregates multiple life-domains (psychological, social, occupational, financial wellbeing, etc.), from domain-specific wellbeing concepts such as *occupational wellbeing* or *work wellbeing* (Wijngaards et al., 2022; P. Warr & Nielsen, 2018; Pandey et al., 2025). Bartels et al. (2019) criticised the tendency to adopt conceptualisations or measures of general wellbeing and “re-tool” them “to fit the work domains” (p. 2) and noted that the broader wellbeing concept does not capture accurately what it means to flourish at work.

Furthermore, researchers often adopt *psychological wellbeing* models and measures, such as M. E. Seligman’s (2012) PERMA model or Ryff’s (1989) Psychological Wellbeing model, as means to conceptualise and assess *employee wellbeing* or *work wellbeing* (Laine & Rinne, 2015; Pandey et al., 2025). Kenttä and Virtaharju (2023, p. 562) critiqued this tendency while noting the “increasing dominance of the psychological vocabulary” in *organisational wellbeing* scholarship.

Compounding this, constructs that are considered components of the work/er wellbeing construct, such as *subjective wellbeing at work* (Magnier-Watanabe et al., 2023), *emotional wellbeing at work* (Salavera & Urbón, 2024), *happiness at work* (Fisher, 2010), *workplace happiness* (Kun & Gadanecz, 2022), and *job satisfaction* (Wartenberg et al., 2023), and on the negative side—*work stress* (Burman & Goswami, 2018) and *occupational burnout* (Demerouti et al., 2021), are sometimes used as proxies for larger wellbeing concepts (though it is often unclear which one), and at other times treated as distinct constructs. For example, Valtonen et al. (2025) observed that some studies conflate *employee wellbeing* with *job satisfaction*, *psychological wellbeing*, and *subjective wellbeing*. Likewise, Schmidt et al. (2008) noted that *satisfaction with work* is often treated as a proxy for *quality of life at work*, while P. Warr and Nielsen (2018) noted that several proxies are used in the literature for *job-related wellbeing*, including *job-satisfaction*, *life satisfaction*, and *job-related affect.*

Further ambiguity arises from the multiple terms that denote high or low levels of work wellbeing, such as *flourishing at work* (Peethambaran & Naim, 2025) *thriving at work* (Kleine et al., 2023), and, on the negative side, *work illbeing* (Jarden et al., 2023) or *suffering at work* (Allard-Poesi & Hollet-Haudebert, 2017), and *languishing at work* (Rautenbach & Rothmann, 2017). In the literature these terms are mostly used as synonymous terms for a variety of broader wellbeing concepts. For example, in one study the term *flourishing at work* has been used to denote *employee wellbeing* (Redelinghuys et al., 2019), and in another paper it depicts *workers’ mental health* (A’yuninnisa et al., 2024).

Kelley (1927) coined the term *jingle–jangle fallacy* to characterise two common conceptual errors, proposing that a jingle fallacy arises when a single label is used for different constructs, whereas a jangle fallacy occurs when different labels are used for what is essentially the same construct. As seen in the depiction above, both types of errors seem to feature in the work/er wellbeing literature.

Surprisingly, only few scholars acknowledged the abundance of terms and the terminological ambiguities. Martela (2025) noted that multiple constructs are used to examine *employee wellbeing* and some have developed largely in isolation from one another. Fisher (2014) observed within the work scholarship, “a great number of concepts might be construed as belonging to the family of wellbeing constructs” (p. 14). Both authors noted that this state of terminological tangle has severe ramifications for research. It cascades into the definitions of these terms, and into the scales developed to measure these constructs, and undermines the ability to compare findings across studies, thereby constraining the development of cumulative science and high-level theorising.

## 3. Work/er Wellbeing: Definitions in Disarray

Alongside the proliferation of ambiguous concepts described above, authors have noted that researchers struggle to reach a consensus on the definitions of these terms. For example, with regard to *employee wellbeing*, Pradhan and Hati (2022, p. 387) remarked that its definition remains “unclear and unresolved”, while Pandey et al. (2025) identified six partially converging and diverging definitions of the construct. Similarly, Valtonen et al. (2025) criticised both the absence of an accepted definition of the term and the heterogeneity of its measurement tools, which typically reflects researchers’ own interpretations of the concept, ranging from scales that assess depression and anxiety, to psychological wellbeing, or job satisfaction.

Similar concerns have been raised about other constructs:

- Saraswati et al. (2019) argued that the term *occupational wellbeing* has not been fully integrated into occupational therapy practice, partly because of difficulties surrounding its conceptualisation and definition.
- Regarding the term *quality of life at work*, in their review of 17 studies, Schmidt et al. (2008) observed that there is no consensus on the meaning of the concept.
- With respect to *wellbeing at work*, Schulte and Vainio (2010) noted that numerous definitions exist, and they vary by discipline. Fisher (2014) observed that it has been operationalised in a variety of ways.
- In relation to *work wellbeing*, Jarden et al. (2023, p. 2) commented that “theoretical models and definitions are varied and usually from a Western perspective”, while De-Neve and Ward (2025) noted that the concept is regarded as vague and ill-defined, thereby hindering its scientific development and its practical implementation.
- Danna and Griffin (1999) described *wellbeing in the workplace* as conceptually vague.
- Focusing on *worker wellbeing*, Wijngaards et al. (2022) noted that the multitude of conceptualisations and measurement approaches makes it difficult for researchers to select and justify their chosen research strategies.
- Finally, Kenttä and Virtaharju (2023) examined the concept of *organisational wellbeing* and questioned whether “a single wellbeing vocabulary be all-encompassing” (p. 560) and opined that a a concept “cannot be accurate, simple, and comprehensive at the same time” (p. 560).

Table 1 presents a sample of concepts and their definitions to illustrate the extent of the conceptual disarray. It is noteworthy, however, that most papers do not define the work-related wellbeing construct being examined.

A close review of the concepts and definitions presented in Table 1 highlights some intriguing points. First, some terms are conceptualised in different ways (for example *employee wellbeing*). Second, definitions vary in what they refer to and in their levels of abstraction. Some definitions state what the term means (for example Huang et al.’s (2007) exploration of *Quality of work life*), while others highlight the components of the construct, (see Fisher’s (2014) definition of *wellbeing at work*), or refer to its subdomains (for example, Pandey et al.’s (2025) *employee wellbeing* definition), or examine the factors that shape wellbeing (see Martela’s (2025) *employee wellbeing* description). Lastly, some of the definitions also use other, similarly vague terms, to explain the concept they aim to unpack (for example Zheng et al.’s (2015) *employee wellbeing* definition).

Importantly, most descriptions draw on Positive Work and Organisations scholarship to define the concepts, with references to hedonia and eudamonia as key components of wellbeing repeatdy featuring in these definitions (for example Jarden et al.’s (2023) *worker wellbeing* definition, and Valtonen et al.’s (2025) *employee welbeing* conception).

Taken together, the abundance of concepts in this body of literature, the heterogeneity in how each is defined, the lack of precision, and the varying levels of abstraction and categorisations all suggest that work/er wellbeing research has reached a critical point of fragmentation and conceptual disarray, and is in pressing need of clarification and unification. In the next section, we turn to examine the underlying causes or sources that led to this state of affairs.

## 4. Tracing the Sources of the Terminological and Conceptual Tangle in Work/er Wellbeing Scholarship

What underlies the proliferation of concepts, ambiguous terminology, and inconsistent definitions? Only a few authors have sought to examine this issue, and their discussions have largely centred on three aspects: the legacy of historical research traditions, the challenges arising from interdisciplinary work, and difficulties associated with the overarching construct of wellbeing.

### 4.1. Legacy Terminology

A key source of the current terminological ambiguity, conflation, and clutter in our view stems from three historical research streams (outlined below), which continue to run in parallel today, each with its own vocabulary. These streams developed in step with industrial, psychological, technological, and social transformations, and can be categorised into three broad phases:

- **Phase 1: A focus on occupational safety and risk mitigation (1900–1950):** During this early period, worker wellbeing was predominantly construed from a preventative perspective, aiming of mitigating workplace injuries and occupational illnesses during a time of rapid industrial expansion. Legislative reforms and organisational practices centred on physical safety, limiting exposure to hazards and unsanitary conditions through policies and statutory safeguards. Institutions such as the International Labour Organisation (est. 1919) and the International Commission on Occupational Health (est. 1906) helped formalise occupational health as a risk control and hygiene-oriented field, promoting safe work standards and the systematic recording and notification of occupational accidents and work-related diseases (Greenfield, 2020; Grut, 1951).

Over subsequent decades, this orientation developed into the contemporary Health and Safety paradigm implemented across numerous organisations. It encompasses the policies, practices, and systems designed to protect and promote employees’ health and safety at work, principally by preventing accidents, injuries, and occupational diseases (Hughes & Ferrett, 2011). Labour movements and advances in scientific knowledge shaped modern safety regulation (Aldrich, 1997), which require employers to provide safe working environments, conduct risk assessments, and introduce preventive measures. Within this framework, worker wellbeing is often equated with physical health, and defined as the absence of injury or illness, and many organisations still operate with this bounded conception.

- **Phase 2: Organisational psychology—a remedial focus (1960–1990):** This period marked a shift toward organisational psychology with a distinctly remedial orientation, extending the wellbeing concept to encompass psychological phenomena such as stress, burnout and workaholism. Researchers investigated the mental load of modern work, and interventions included employee counselling. The concept of wellbeing shifted toward the management of psychological distress, moving beyond physical aspects (Wallace, 2022).

Two strands of research and practice rose to prominence:

- **Occupational Health Psychology** (OHP) is an applied field focused on improving working life and safeguarding worker wellbeing. It addresses stress and burnout, safety behaviours and culture, supporting healthier work–life balance, and mitigating or treating the consequences of aggression and bullying at work (Quick, 1999; Schonfeld & Chang, 2017). Today OHP remains explicitly health-centred, prioritising workers’ wellbeing, safety, and health, with interventions typically aimed at reducing workplace stressors and risks.
- **Business (Organisational) Psychology** is the science and practice of improving working life and organisational functioning. Key themes include personnel selection and recruitment, training and development, performance appraisal, leadership development, change and restructuring, and team effectiveness (McKenna, 2020). While intersecting with OHP, it has a broader remit and commonly aims to enhance outcomes such as productivity, retention, and efficiency.

Due to the dominance of these approaches, psychological work-related conditions and disorders attracted the attention of researchers and practitioners (Maslach et al., 2001), and organisations adopted policies and practices that address factors that undermine employees’ wellbeing (such as overwork, occupational risk, organisational change, and bullying).

Today, both disciplines sit alongside the Health and Safety model and retain a largely curative orientation to wellbeing focusing on compromised work wellbeing, and the factors that can erode it. At the same time, they integrate several aspects of wellbeing, including physical wellbeing (seen as the absence of illness or injury), and psychological wellbeing (assessed through the absence of occupational psychological disorders), and examine organisational factors that impinge on wellbeing such as organisational culture, policies, structures, job design, and the work-life interface (Quick, 1999; McKenna, 2020).

- **Phase 3: The holistic and positive wellbeing era (1990–present):** Contemporary approaches adopt multidimensional models that span physical, psychological, social, and economic research. Current thinking draws heavily on Positive Work and Organisations—an interdisciplinary field synthesising Positive Psychology with Management scholarship, defined as the study and application of psychological processes that foster employee and organisational flourishing (Warren et al., 2017). The overarching term Positive Work and Organisations (or Positive Work and Organisational Psychology) is a relatively recent label that integrates three closely related subfields:
- **Positive Organisational Psychology** (POP; Donaldson & Ko, 2010) is concerned with the scientific study of positive subjective experiences in the workplace. Its primary focus is on individual employees and the interface between employees and their organisations, with the aim of developing applications that enhance individuals’ quality work life. Topics include occupational wellbeing, positive leadership, job satisfaction, work engagement, motivation, positive relationships, and meaningful work.
- **Positive Organisational Behaviour** (POB; Luthans, 2002) is defined as “the study and application of positively oriented human resource strengths and psychological capacities that can be measured, developed, and effectively managed for performance improvement in today’s workplace” (Luthans, 2002, p. 59). It emphasises individual-level positive psychological states and resources that relate to wellbeing or performance, such as strengths, hope, optimism, resilience, and prosocial behaviour.
- **Positive Organisational Scholarship** (POS; Cameron et al., 2003) is defined as “the study of that which is positive, flourishing, and life-giving in organisations” (Cameron & Caza, 2004, p. 731). It focuses on organisational characteristics and processes, and on their implications for employees and organisations, including phenomena such as organisational virtuousness, psychological safety, diversity, equality and inclusion, positive deviance, and appreciative cultures (Cameron et al., 2003). Its core concern is the achievement of work-related outcomes through positive means.

As these definitions illustrate, the three subfields overlap to some degree, which has led to conceptual ambiguity. To reduce misunderstandings, Warren et al. (2017) proposed the umbrella term Positive Work and Organisations (PWO) to encompass all three areas. The overarching purpose of PWO is “to encourage dialogue across subfields and serve as a clearinghouse for best practices in positive psychology theory building and practice” (Donaldson et al., 2019, p. 114). Definitions of work wellbeing have therefore shifted from an exclusive harm-avoidance or remedial lenses to more holistic accounts (Kenttä & Virtaharju, 2023; Wallace, 2022).

Despite this progress, for nearly three decades the conceptualisation of work/er wellbeing remained ambiguous and contested (Martela, 2025). Much of the research in Positive Work and Organisations borrowed from broader Positive Psychology wellbeing frameworks and measures, resulting in scarce constructs tailored specifically to work. This relative stagnation may reflect the maturation trajectory of wellbeing as a concept (Pipera & Fragouli, 2021). Recent advances in conceptualising wellbeing (see below) have prompted a renewed interest in its workplace expression, spurring fresh conceptual analyses and reviews (Martela, 2025; Bautista et al., 2023; Pandey et al., 2025) (see details below).

As these historical trends unfolded, the concept of work/er wellbeing underwent changes in its meaning, definitions, and associations, a process known as semantic drift (Stavropoulos et al., 2019). Although such drift can result in conceptual replacement, this did not occur in this instance. In fact, the three traditions seem to operate side by side in contemporary practice, though in some organisations they are beginning to converge into integrated wellbeing strategies that couple psychosocial risk management and job design with growth-oriented practices and individual supports (Aubouin-Bonnaventure et al., 2021). In scholarship, however, they proceed both in parallel and in concert: researchers draw selectively across frameworks, mix constructs, and apply heterogeneous measures (Jarden et al., 2023). We argue that this simultaneous separation and blending contributes to persistent terminological and conceptual imprecision in the work/er wellbeing literature. For example, the varied *quality of work* terms have emerged from the remedial scholarship and practice, while the *health* related terms emerged from the health and safety tradition. It is also noteworthy that these legacy terms continue to evolve. However, they often do so without adopting the more mainstream labels or clearly differentiating between those that refer to health, wellbeing, or quality of life.

### 4.2. Interdisciplinary Challenges

A second source of the conceptual clutter and ambiguity according to Wassell and Dodge (2015) can be attributed to the multidisciplinary nature of work/er wellbeing scholarship, since “different fields of study use different vocabulary and models to understand the complexity of wellbeing” (p. 98).

Contemporary work/er wellbeing research indeed draws on a wide range of disciplines, including various branches of Management and Psychology, as well as Philosophy, Health, Economics, Policy studies, Neuroscience, Education, Social Work, and Environmental studies, among others. This multidisciplinary interest has generated an abundance of constructs, synonymous terms, varied definitions, and diverse interpretations of wellbeing, its dimensions, and its components, which speaks to the richness of the field. At the same time, it exposes the underdevelopment of one of the key foundations of interdisciplinary work—**a common language**, giving rise to substantial misunderstandings and recurring critique.

In their examination of the processes involved in the emergence of new interdisciplinary scientific fields, Lyall et al. (2011) distinguished between three approaches to integrating knowledge across disciplines:

- **Multidisciplinary research:** In this approach, researchers from different disciplines engage with a shared research problem while largely retaining their own disciplinary lenses. The contributing disciplines remain separate, offering parallel inputs to the research question without substantial integration of their methods or theoretical frameworks.
- **Interdisciplinary research:** This approach is more integrative, involving the deliberate amalgamation of theories, concepts, and methods from multiple disciplines. Researchers collaborate across disciplinary boundaries to co-create new frameworks and methodological approaches, with an emphasis on synthesising and unifying diverse perspectives.
- **Transdisciplinary research:** This category represents the highest level of integration, extending collaboration beyond academic disciplines to include non-academic stakeholders such as policymakers and practitioners. The focus is on addressing complex, real-world problems, rather than remaining confined to discipline-based theoretical concerns.

Each of these approaches embodies a distinct degree of collaboration and integration across different fields of study, and many interdisciplinary scholarships move from one approach to the next as they mature, and as the amalgamation between the parent disciplines becomes robust and applied more widely.

We maintain that Positive Work and Organisations now regarded as a leading perspective in this area, has advanced well beyond multidisciplinary research, as there are strong indications of interdisciplinary work and new theoretical frameworks and methods emerging (see reviews by Van Zyl et al., 2023; Meyers & Rutjens, 2022; Donaldson et al., 2022; Sonnentag et al., 2023). There are also signs that its applications are being adopted outside the academic sphere—indicating a transdisciplinary approach (see Nielsen et al., 2017; Saleh, 2025).

Lyall et al. (2011) emphasised that a key stage in the development of new interdisciplinary fields is the creation of a **shared language**, which is crucial for sustaining the discipline’s growth. When this stage is absent or partially completed, the differing ‘languages’ of the disciplines mean that, even when partners believe they have achieved mutual understanding, it may later become evident that their interpretations diverged—by which point difficulties in coordinating strands of work may already have arisen. Consequently, the same term may be used to denote different concepts across disciplines, and different terms may be used to describe the same construct—a classic jingle jangle fallacy (Kelley, 1927).

We argue that this characterisation aligns closely with the current jumble of terminology in the work-related wellbeing field and may offer an explanation as to how this state has evolved. The fact that only a small number of authors have remarked on this issue suggests that there has been no substantive discussion of language and terminology in this sphere, which we hope to instigate.

### 4.3. The Construct of Wellbeing

A third, and arguably central, source of the current conceptual clutter concerns the developmental status of the overarching construct of wellbeing. Pipera and Fragouli (2021) observed that overall wellbeing is a complex, multidimensional concept that is difficult to define and measure, and that this lack of clarity is reflected in related work-focused constructs. We agree with this observation and note that the concept of wellbeing has seen similar challenges to those reviewed above and likewise attracted fierce critique.

Several authors noted the multitude of synonymous terms used in the literature to represent wellbeing, including *health*, *quality of life* and *wellness* (Pincus, 2024; Roscoe, 2009). Furthermore, and similar to the conundrum reported in work/er wellbeing scholarship, in much of the research, terms that represent components of wellbeing, including *subjective wellbeing*, *emotional wellbeing*, *happiness*, *utility* and *life satisfaction* are often conflated with the term wellbeing or used as proxies (Pincus, 2024). Terms that denote high or low levels of wellbeing including *optimal wellbeing*, *flourishing*, *thriving*, *prosperity*, and on the low side, *distress*, *suffering*, *struggling* and *ailing*, are also used interchangeably with wellbeing (Lomas et al., 2023).

Pincus (2024) argued that the *wellbeing* literature has become saturated with a proliferation of constructs, dimensions, components, and subcomponents. These core terms carry multiple meanings and are employed in contested, and at times conflicting ways, which makes establishing common ground difficult. Lomas et al. (2023) considered the concepts of *wellbeing* and *health* particularly difficult to disentangle, since contemporary discourse variously treats them as synonymous terms, presents health as a subset of the broader notion of wellbeing, or vice versa, or depicts them as partially overlapping, with the boundaries between them often left unclear.

Analogous to the conceptual ambiguity surrounding work-related wellbeing constructs, the lack of a shared definition of the broader term—*general (or overall) wellbeing*, has also been the subject of substantial critique. Lomas et al. (2023) noted that, despite extensive scholarly work, the question of how wellbeing should be defined remains largely unresolved. Consequently, definitions of wellbeing have become “blurred and overly broad” (Forgeard et al., 2011, p. 81). Thomas (2009, p. 11) contended that wellbeing is “intangible, difficult to define, and even harder to measure,” while Pollard and Lee (2003, p. 60) characterised it as “a complex, multi-faceted construct that has continued to elude researchers’ attempts to define and measure.” Pincus (2024) observed that the field is “highly fragmented with muddled theoretical and operational definitions” (p. 1542). Other reviews have catalogued the diverse ways in which wellbeing has been defined (Blount et al., 2020; Oliver et al., 2018; Linton et al., 2016; Roscoe, 2009), but their findings offer little reassurance to those seeking greater clarity.

A similar line of critique has been directed at the concept of *wellness*. Roscoe (2009, p. 216) observed that “despite significant attention to *wellness* in the literature, there is surprisingly little consensus on the definition of the construct”. Blount et al. (2020) argued that *wellness* is conceptualised differently across professions, hence complicating its application. In the helping professions, *wellness* underpins therapeutic philosophy, codes of practice, and policies, and therefore the absence of a shared definition has direct implications for the care provided to clients.

As seen earlier, these terminological ambiguities seem to have cascaded into work-related *wellbeing* concepts as well as into other life-domains of wellbeing, where constructs such as social, psychological, physical, and financial wellbeing are likewise characterised by multiple synonymous labels and imprecise definitions and interpretations. For example, in reference to *financial wellbeing* Garcia-Mata and Zerón-Félix (2022) identified 18 definitions of the term, while Riitsalu et al. (2023) highlighted the lack of consensus around a definition.

Intriguingly, the conceptualisation of the domains of *overall wellbeing* has become in itself an area of ambiguity within the wellbeing scholarship. Pincus (2024, p. 1544) argued that the field has been gripped by an “arms race of expanding domains. In the 1970s, wellbeing models included an average of four domains; this increased to an average of five in the 1980s and 1990s; since 2000, the average number of domains has again increased to seven domains, with some entrants proposing 12 and up to 17 domains of wellbeing”. Most frameworks include physical, social, and mental dimensions; around 60% also incorporate spiritual, intellectual, occupational, financial, and environmental dimensions, while only a minority address additional, more nuanced facets such as growth, creativity, identity, and autonomy.

This indicates that there is no consensus on how many life domains the *overall wellbeing* concept encompasses with some authors working with broader terms, while others focus on narrower concepts.

This lack of conceptual clarity has also extended into the work-related wellbeing sphere. Some authors treat constructs such as *work wellness* as subdimensions of an overarching wellness construct (Wicken, 2000). In contrast, others regard concepts such as *employee wellbeing* as broad term that incorporates several subdomains (*social, financial, physical*, and *psychological wellbeing*) (Pipera & Fragouli, 2021).

An additional cause of the terminological jumble is the lack of distinction between terms that refer to different aspects of the wellbeing concept. Pincus (2024, p. 1542) defined these as “category errors” and argued that the broader *wellbeing* literature is “awash” with such esrrors, including failure to distinguish between the overarching wellbeing construct and domain-specific forms of wellbeing, conflations of causes and effects, traits and states, endogenous psychological variables and exogenous variables, applying psychological wellbeing models as proxies for other domains, and confusion between components and predictors of wellbeing—that is, between what wellbeing *is* and the factors that shape or promote it.

We concur with this assessment and further propose that additional category errors arise from insufficient distinctions between broad accounts of the core components of wellbeing (for example the hedonia/eudaimonia distinction), and theoretical or empirical models of work/er wellbeing, which elaborate and operationalise these theories into specific components (for example, the PERMA+4 model (Donaldson et al., 2022), and measures of work/er wellbeing, which frequently cut across these levels (Cooke et al., 2016; Jarden et al., 2023).

One way to resolve these ambiguities is to create a conceptual map of the terrain that shows the hierarchical relationship and points of convergence and divergence between these terms, which we attempted to offer below.

In conclusion, the current conceptual disarray in work/er wellbeing scholarship appears to stem from several interrelated sources: the coexistence of three largely parallel research strands its interdisciplinary challenges, and lack of agreement on terminology, and ambiguities in the overarching concept of *wellbeing* that have cascaded into the work domain. Encouragingly, recent advances in the conceptualisation of wellbeing more broadly offer promising pathways for resolving these issues, and it is this point that we explore next.

## 5. Reconceptualising Work/er Wellbeing: Towards an Integrative Meta-Framework

Despite growing recognition that work/er wellbeing scholarship is messy (Wijngaards et al., 2022) and characterised by a dense terminological and conceptual tangle (Makikangas et al., 2016), relatively little systematic effort has been made to untangle it. While authors have commented on the proliferation of overlapping constructs, the lack of clear definitions, or the ambiguity surrounding existing terms, these critiques have seldom been accompanied by substantive proposals for conceptual clarification or integration. Instead, most contributions either draw on existing definition, introduce yet another definition—often with limited engagement with prior usage, or proceed without offering any explicit definition, thereby perpetuating the underlying ambiguity.

The few authors who attempted to offer ways to disentangle the conceptual maze firstly differentiated between *work-related health* and *wellbeing* constructs (Danna & Griffin, 1999), arguing that wellbeing is the broader of the two, and that health encompasses both physical and psychological indicators.

Lomas et al. (2023) also attempted to distinguish between the broad *health* and *wellbeing* concepts. They argued that when the two terms are not used synonymously, *wellbeing* appears to refer primarily to subjective aspects of the person (how one perceives their own state—physically, mentally, financially, careerwise, etc.), whereas *health* tends to denote more objective aspects (how well an aspect of life is operating based on an objective assessment). On this basis, they proposed that a useful way to differentiate *health* and *wellbeing* is via a subjective–objective distinction, and accordingly, they defined *health* as “the relative attainment of a personal objective state of quality” (p. 8).

While differentiating the objective and subjective dimensions is valuable, the authors did not indicate how this distinction should be operationalised in empirical research or in practice. A further difficulty with this binary concerns constructs that involve both objective and subjective elements. The authors did not provide guidance on how such constructs should be classified.

In an effort to disentanle other concepts, Wijngaards et al. (2022) proposed several useful distinctions between *overall wellbeing*, *worker wellbeing*, *employee wellbeing*, and *work wellbeing*:

- *Overall wellbeing*: The broadest category pertaining to the *global wellbeing* of an individual.
- *Worker wellbeing*: Describes the overall wellbeing of working people.
- *Employee wellbeing*: Refers to the *overall wellbeing* of those employed by organisations.
- *Work wellbeing*: A narrower term that is specific to the work domain.

One point that remains unclear in Wijngaards et al.’s (2022) analysis is whether the term *overall wellbeing* aggregates several domains (physical, psychological, social, financial, and work wellbeing).

However, there are also conflicting claims regarding the hierarchical relationship between these terms. Zheng et al. (2015) argued that overall wellbeing is an inadequate proxy for employee wellbeing, whereas Pipera and Fragouli (2021) contested this view, proposing that employee wellbeing should be conceptualised as encompassing both work-related and non-work-related physical, mental, and social states.

Below we attempt to resolve some of these points by offering a hierarchical structure that classifies how the varied work/er wellbeing constructs relate to one another.

### 5.1. Mapping the Key Concepts That Feature in Work/er Wellbeing Scholarship

**Figure 1:**
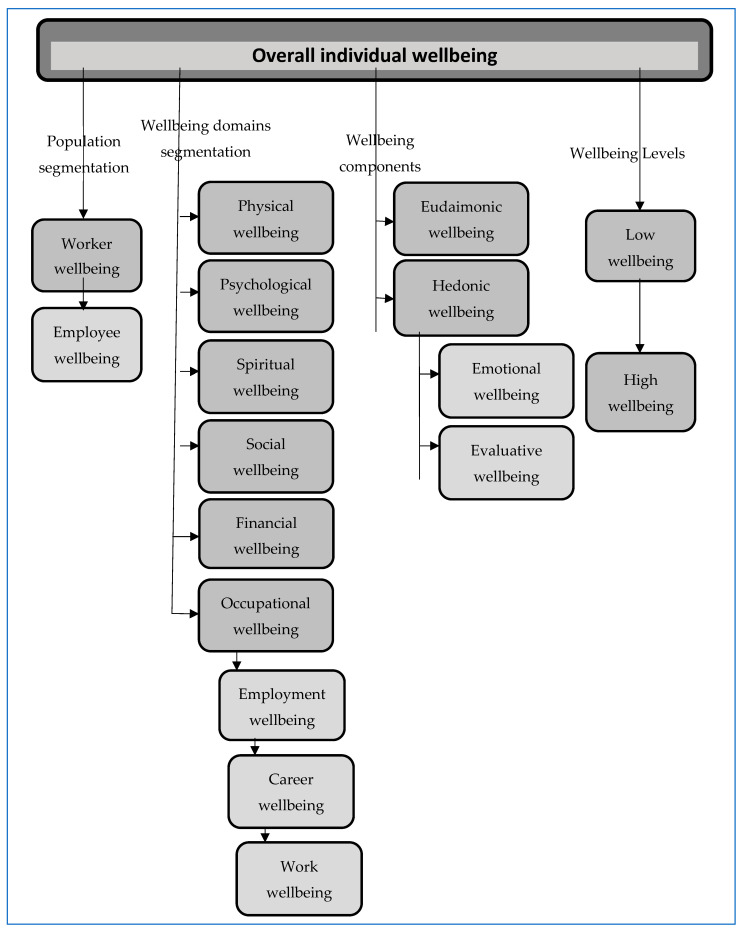
A conceptual map of key work/er wellbeing terminology.

The suggested conceptual map (shown graphically in Figure 1 below) is hierarchical, progressing from the broadest to the specific constructs. It mainly draws on Positive Work and Organisational scholarship due its interdisciplinary scope and its widespread contemporary use, applying its core conceptualisation (see details below) to clarify how the terms relate to one another.

The starting point for the conceptual map is to clarify the facets of wellbeing included in the map (as well as some that are not included) and define these terms. Table 2 presents a glossary of these terms and their proposed definitions, meanings and applications in the work-related wellbeing scholarship.

**Figure 2:**
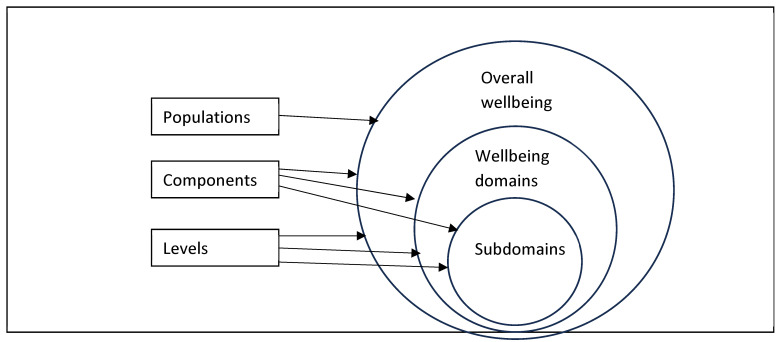
Specifying the association between the map’s components.

The conceptual map breaks down the overarching wellbeing concept into domains, populations, components, and levels. Figure 2 clarifies the associations between the map’s components, indicating that the organising logic of the map is that its elements are hierarchically nested, moving from broader structures (overall wellbeing), to life domains (physical, psychological, social, etc.) and to sub-domains (where these exist), with Figure 1 focusing solely on the occupational domain. As the map indicates, in examining work-related domains, occupational wellbeing is the broadest term, and work wellbeing is the most narrowly defined. The other components of the map: populations, components and levels can be applied to overall wellbeing, to domains and to sub-domains, though we note that in the work-related literature the reference to particular populations tend to be framed in terms of their overall wellbeing.

We note that the term “occupational wellbeing” is proposed here as a container category encompassing wellbeing in relation to employment, careers, and work. This avoids the repeated use in the wider literature (and in this paper) of the term “work-related wellbeing concepts”, which is imprecise, as it refers narrowly to work rather than also capturing careers or employment.

### 5.2. Defining the Key Terms in the Work/er Wellbeing Scholarship

Within Positive Psychology scholarship, wellbeing has come to denote a broad conception of a “life well lived”, that is, the extent to which an individual leads “a good life” (Hart, 2020). However, this broad and somewhat abstract characterisation requires further specification regarding what “a good life” entails. In a recent paper, Lomas et al. (2023, p. 8) defined it as a continuum that indicates “the relative attainment of a personal subjective state of quality”. This definition suggests that wellbeing reflects a state, and when referring to overall wellbeing, it aggregates specific life domains to offer an overall assessment. Hence *overall wellbeing* can be defined as “the quality of one’s personal subjective state across the physical, mental, social, and spiritual dimensions of existence” (Lomas et al., 2023, p. 11). The authors defined “quality” as a “sense of a phenomenon being good or valuable” (p. 8), suggesting “goodness” as an approximate synonym, which involves an appraisal of value or excellence.

We endorse this definition and, in Table 3 below, we have sought to apply it to the various work-related wellbeing constructs depicted in the conceptual map in Figure 1 above. We also highlight (where relevant) synonymous terms.

This exercise helps to resolve some of the conceptual ambiguity noted in the earlier section by drawing clearer distinctions between key terms. For instance, *worker wellbeing* is conceptualised as continuum that reflects a worker’s relative attainment of a personal subjective state of quality across key life domains, whereas *work wellbeing* is conceptualised as continuum that reflects the relative attainment of a personal subjective state of quality in their current work domain, hence the two definitions clearly highlight the differences between these terms.

### 5.3. Components of Wellbeing

Positive Psychology encapsulates wellbeing as a concept comprising two elements: **feeling good** and **functioning well** (Huppert & So, 2013; Hart, 2020; Keyes & Annas, 2009; Deci & Ryan, 2008). These dimensions are grounded in two major philosophical traditions (Huta & Waterman, 2014):

- **Hedonia,** characterised by the pursuit of pleasure, and
- **Eudaimonia,** concerned with optimal functioning across life domains.

The hedonia–eudaimonia distinction can be traced back to Aristotle’s view that wellbeing arises not only from meeting basic needs and desires, but also from performing well in diverse life domains, enacting virtues, engaging in morally worthy pursuits, and realising one’s potential (Huta, 2016). Aristotle used the term eudaimonia to describe a way of life that enables the attainment of an elevated state of quality and value (Huta, 2020).

Hedonia, on the other hand, is commonly equated with happiness. In traditional Greek philosophy, hedonism was viewed as a central component of the good life, reflected in a cognitive and emotional state of contentment (Ryff et al., 2021). It is also understood as an outcome of eudaimonia—how well a person is functioning (Huta, 2016).

With the emergence of Positive Psychology, research on both concepts has expanded substantially, revealing a medium to strong association between them (M. Seligman, 2018). There is also an increasing recognition that “happiness constitutes a subset of wellbeing” (Lomas & VanderWeele, 2023, p. 3), and that the relationship between hedonia and eudaimonia is bi-directional: an individual’s level of functioning influences their happiness, while their level of happiness, in turn, shapes their functioning (Sheldon & Lyubomirsky, 2021; Pavot & Diener, 2004).

Building on these insights, a growing consensus within Positive Psychology holds that overall wellbeing comprises both hedonic and eudaimonic components, and that each is essential for a fulfilling life (M. E. Seligman, 2012). This conception is currently being applied in numerous disciplines, including the work/er wellbeing scholarship.

This leads to the concept of happiness. In the academic literature, happiness is commonly described as hedonic or subjective wellbeing (SWB) (Diener, 2000), which consists of two principal components:

- **Cognitive component**: An evaluation of one’s overall life satisfaction; a subjective assessment of one’s contentment across key life domains such as health, relationships, work, and finances.
- **Emotional component**: This reflects the frequency with which individuals experience positive emotions (e.g., excitement, joy, love) and negative emotions (e.g., fear, anger, disgust) over a given period.

Subjective wellbeing is often expressed using the following formula:

SWB = Satisfaction with life + Positive affect − Negative affect

By definition, individuals with high subjective wellbeing tend to be satisfied with their lives, experience positive emotions frequently, and negative emotions infrequently (Diener, 2000). The experience of positive emotions is therefore central to happiness, which is why happiness is frequently treated as synonymous with hedonia (Huta, 2020).

Lomas and VanderWeele (2023) defined happiness as a “mental experience of quality” (p. 11), thereby conceptualising it as a psychological phenomenon that involves a cognitive judgement, accompanied by an emotional response. They further describe satisfaction with life as “evaluative happiness” (p. 12).

We do not fully endorse this definition, as we consider that it is too closely worded to the wellbeing definition, and therefore likely to generate further confusion. Instead, we propose that *overall happiness* may be defined as a continuum reflecting an individual’s overall life evaluation and emotional experience. This definition can be applied to overall happiness, spanning across key life domains, or to happiness within a specific domain, such as work, relationships, finances, etc. In Table 4 below, we applied this definition to the components of work-related wellbeing concepts identified in the literature.

### 5.4. Levels of Wellbeing

In this section, we unpack terms that denote a person’s level of wellbeing. Notably, relatively few authors have attempted to define these concepts explicitly:

- ***Flourishing*** is defined as a desired state of overall wellbeing, indicating the relative attainment of a condition where all aspects of a person’s life are positive, and their living environments are conducive (Lomas & VanderWeele, 2023).
- ***Thriving*** is conceptualised as a desired state of overall wellbeing attained despite facing challenging or inhospitable contexts and circumstances (Lomas et al., 2023).
- ***Languishing*** denotes a lower level of wellbeing located around the middle of the wellbeing continuum (Keyes, 2002). It can be defined as an ambivalent condition characterised by a relative absence of positive states of quality, without the presence of distinctly low-quality states.
- ***Struggling*** is a lower state of wellbeing, described as an ambivalent condition in which individuals attain some personal states of quality while simultaneously experiencing personal states that lack quality (Lomas et al., 2023).
- ***Illbeing*** (floundering or ailing) can be understood as an undesirable condition characterised by the absence of personal states of quality alongside the presence of personal states that lack quality (Venning et al., 2013).

These can be applied to refer to a person’s overall wellbeing across their key life domains, or within a specific life domain such as career or work. The key point is that these terms should be used only when authors are referring to specific gradations of wellbeing, rather than as proxies for the broader concept of wellbeing.

## 6. Operational Implications

Our analysis, conceptual map, and unified definitions have several direct implications for how work-related wellbeing is operationalised in research and practice.

First, they provide a structured basis for construct selection and specification. Researchers can use the map to decide whether their focal construct is a population-segmented overall wellbeing construct, or a domain-specific construct. This distinction should be reflected in study aims, hypotheses, and in measurement choices. Concretely, when the research question concerns wellbeing *in the current work context*, measures should be selected or adapted to target work wellbeing (and its hedonic/eudaimonic components) rather than importing context-free or psychological scales as proxies. Conversely, when examining broader outcomes, employee/worker wellbeing (as an aggregate across domains) is the more coherent target construct. For instance, in a cross-sectional study examining whether a cluster of work-related conditions predict wellbeing, researchers should specify what is being assessed as wellbeing: work-wellbeing, employees’ overall wellbeing, their psychological wellbeing or other domains of wellbeing (financial, physical, etc.), using the map to distinguish and define more precisely the target variable.

In practice, the same logic supports clearer programme design: organisations can distinguish between initiatives aimed at improving employees’ *overall* wellbeing versus those targeting *work-domain* wellbeing.

Second, the framework strengthens measurement alignment and comparability. A persistent issue highlighted in this paper is the reliance on proxies, in particular the use of life satisfaction or job satisfaction as proxies for work-wellbeing or for overall wellbeing. Another commonly used proxy is the use of psychological wellbeing measures as means to assess the overall wellbeing of employees or their work-wellbeing. The map provides a practical guide for selecting measures that best match the intended concept. When proxies are unavoidable, it supports a more nuanced and defensible choice by ensuring the proxy follows the same underlying logic as the target construct. For example, using job satisfaction as a proxy for work-wellbeing rather than using life-satisfaction. When multiple indicators are employed, the map can also inform the development of composite operationalisations that retain conceptual coherence. Over time, this explicit “construct mapping” should enhance cross-study comparability and evidence synthesis by making studies on par with each other at the level of construct, domain, and component.

Thirdly, the framework has implications for intervention targeting and evaluation. Because the map separates domains and components, it helps translate diagnostic data into appropriately matched levers. For example, low work happiness indicates different organisational actions than low eudaimonic wellbeing, which call for different interventions. Similarly, distinguishing employment wellbeing from work wellbeing clarifies which stakeholders and systems should be engaged. In evaluation terms, the definitions set clearer success criteria: programmes should be assessed against the construct they intend to shift, rather than relying on convenient but conceptually mismatched proxies. This strengthens the translation of evidence into practice by making it explicit what changed, in which domain, and through which component of wellbeing.

We anticipate that this work will also stimulate new theoretical models of work-related wellbeing, another area characterised by a comparable conceptual tangle that has constrained cumulative progress.

Further work is also needed on the interdisciplinary front. Closer collaboration across disciplines is required to enable transdisciplinary translation through the co-production of a usable taxonomy.

## 7. Conclusions

This paper set out to address a persistent, field-limiting problem in work/er wellbeing scholarship: a proliferation of partially overlapping terms, inconsistent definitions, and widespread proxy use that collectively obstruct cumulative theory-building and the translation of evidence into practice. It positioned this “conceptual jungle” not as a superficial terminological nuisance, but as a structural barrier to interdisciplinary integration and measurement coherence. This paper’s core contribution is therefore conceptual: it clarifies *what* is being discussed when scholars and practitioners invoke work/er wellbeing language, *why* confusion has arisen historically, and *how* a more ordered vocabulary can be established via mapping and definitional refinement.

This paper firstly documented the scope of terminological clutter: the multiple labels used sometimes as synonyms and sometimes as distinct constructs. It then showed how definitions remained unclear, with many studies providing no definitions, and others offering definitions that vary in abstraction, which further encourages construct conflation and incompatible operationalisations.

The analysis then traced the sources of the tangle, highlighting the co-existence of legacy terms, interdisciplinary language gaps, and the contested nature of the overall wellbeing concept, including category errors. This diagnosis motivates this paper’s proposed remedy: an explicit classification system that reduces category errors and makes it easier to select, define, and measure constructs in defensible ways.

The reconceptualisation section advanced an integrative, hierarchical meta-framework and conceptual map that distinguishes between context-free overall wellbeing concepts and wellbeing specific domains, terms that refer to varied populations, concepts that describe the components of wellbeing, and expressions that depict different levels of wellbeing. This paper adapts recent definitional advances in wellbeing scholarship to the work domain, yielding clearer definitions for key work-related terms.

Ultimately, the central claim of this paper is that progress in work/er wellbeing science is currently constrained less by a shortage of studies than by a shortage of shared meaning. By “cleaning the lens” on work-related wellbeing concepts—clarifying domains, components, populations, and levels, and separating wellbeing from its proxies, we provide a foundation for more cumulative theory, more comparable evidence, and more defensible measurement. This is not merely a semantic exercise: conceptual precision is a prerequisite for designing workplaces that can reliably tilt the “double-edged sword” of work towards flourishing rather than harm. The next stage, therefore, is collective adoption of a shared vocabulary and mapping logic, so that scholarship and practice can converge on clearer targets, stronger inference, and more effective wellbeing strategies.

## Figures and Tables

**Table 1:** A sample of work-related wellbeing concepts and their definitions.

Concept	Definition
*Overall wellbeing*	*Personal wellbeing* may be understood as life satisfaction derived from an individual’s perceptions of their health, happiness, and sense of purpose (Litchfield et al., 2016).
*Overall health*	*Overall health* comprises a range of factors, including physical, mental, and social wellbeing, and is not merely the absence of disease or infirmity (Jarden et al., 2023).
*Worker wellbeing*	Chari et al. (2018) conceptualised *worker wellbeing* as a quality-of-life construct, referring to an individual’s health in relation to environmental, organisational, and psychosocial factors associated with work. The authors stressed that *worker wellbeing* should encompass both work and nonwork domains and should be assessed using subjective and objective indicators.
	Jarden et al. (2023) proposed that *worker wellbeing* can be understood as the balance between an individual’s resources and the challenges they face at work. Key components include subjective wellbeing, eudaimonic wellbeing, and social wellbeing.
*Employee wellbeing*	*Employee wellbeing* refers to the overall quality of an individual’s experience at work, encompassing their physical, psychological, social, financial, and spiritual wellbeing (Pandey et al., 2025).
	Page and Vella-Brodrick (2009) posited that *subjective* and *psychological wellbeing* should be regarded as key components of *employee wellbeing*.
	P. B. Warr (1990) defined *employee wellbeing* as the overall quality of an employee’s experience and functioning at work. He later described it as employee’s experience and functioning, incorporating both physical and psychological dimensions (P. Warr, 1999).
	Martela (2025) conceptualised *employee wellbeing* as a broad, subjective construct that encompasses the various factors that make work a positive experience for employees.
	Kinowska and Sienkiewicz (2023) observed that *employee wellbeing* is frequently conceptualised in terms of psychological wellbeing, which comprises hedonic perspective, concerned with positive emotions and satisfaction, and a eudaimonic perspective, centred on the realisation of human potential.
	*Employee wellbeing* can be defined as an evaluation of employees’ physical, mental, and social state, encompassing both their work and non-work life experiences (Pipera & Fragouli, 2021).
	Grant et al. (2007) defined *employee wellbeing* as the overall quality of employees’ experience and functioning in the workplace.
	Boxall and Macky (2014) conceptualised *employee wellbeing* as a holistic construct comprising job satisfaction, physical health, mental health, and relationship outcomes.
	Danna and Griffin (1999) described *employee wellbeing* as encompassing employees’ physical and mental health, spanning both their work and broader life experiences.
	*Employee wellbeing* can be defined as how individuals feel and function within their workplaces (Valtonen et al., 2025).
	Zheng et al. (2015) maintained that *employee wellbeing* can be understood as employees’ quality of life and psychological state in the workplace.
*Occupational wellbeing*	*Occupational wellbeing* denotes the sense of meaning and satisfaction that individuals derive from their occupational lives (Doble & Santha, 2008).
	*Ooccupational wellbeing* is understood as the subjective meaning a person ascribes to their occupational life. It comprises several intrinsic needs: agency, accomplishment, affirmation, pleasure renewal, coherence, and companionship (Saraswati et al., 2019)
*Work wellbeing*	Laine and Rinne (2015) observed that most contemporary definitions of *work wellbeing* employ *subjective wellbeing* as a key indicator, referring to individuals’ cognitive and affective evaluations of their lives. *Psychological* wellbeing constitutes another core component of *work wellbeing*, reflecting positive psychological functioning.
*Work/place wellbeing*	De-Neve and Ward (2025) used the term *work wellbeing* interchangeably with *workplace wellbeing* and observed that researchers frequently conflate *workplace wellbeing* with *workplace subjective wellbeing* (*workplace happiness*). They proposed a definition of *workplace wellbeing* grounded in *subjective wellbeing*. *Workplace subjective wellbeing*, in their view, comprises three elements: evaluative job satisfaction, emotional experiences at work, and eudaimonic work wellbeing, that is, experiencing work as purposeful, worthwhile, or meaningful. They further argued that eudaimonia is embedded within subjective wellbeing.
*Workplace wellbeing*	Wallace (2022) conceptualised *workplace wellbeing* as a biopsychosocial construct encompassing physical, mental, and social health.
	According to Leiter and Cooper (2017) *workplace wellbeing* encompasses physical health and comfort, mental health, a predominance of positive over negative affect, and favourable attitudes towards one’s work.
	*Workplace wellbeing*, is “defined as an employee’s subjective evaluation of his or her ability to develop and optimally function within the workplace” (Bartels et al., 2019, p. 3).
	*Workplace wellbeing* refers to employees’ overall experience and functioning, encompassing physical and psychological dimensions (Kinowska & Sienkiewicz, 2023).
*Wellbeing at work*	Fisher (2014) proposed that *wellbeing at work* comprises three components: subjective wellbeing at work (positive judgemnents and the experience of positive and negative affect), eudaimonic wellbeing (engagement in growth-oriented, self-actualising behaviours), and social wellbeing at work (centred on the relational aspects of work).
*Work wellness*	*Work wellness* is an optimal state of living encompassing physical, mental, and spiritual wellbeing. It is a way of life oriented towards optimal health and wellbeing (Klerk, 2005).
	Wicken (2000) described *work wellness* as a component of overall wellness, defined as the process of becoming aware of and making choices toward a more successful existence. It encompassed several dimensions such as social, spiritual, physical, occupational, emotional and intellectual.
*Employee welfare*	Lintong and Bukidz (2024) defined *employee welfare* as a state in which individuals feel happy, healthy, and safe, and have opportunities to develop their potential.
*Quality of work life*	Horst et al. (2014) characterised *quality of work life* as employees’ evaluation of the demands they face and the aspirations they hold in relation to working conditions, remuneration, professional development, work–family and role balance, safety, and social interactions in the workplace.
	*Quality of work life* refers to the favourable workplace conditions and environments that support employees’ welfare and wellbeing (Huang et al., 2007).
*Subjective Wellbeing at Work*	*Subjective wellbeing at work* refers to employees’ emotional experiences at work and their *satisfaction with their work* (Magnier-Watanabe et al., 2023).
*Workplace happiness*	*Workplace happiness* describes employees’ experience of feeling energised and enthusiastic about their work, perceiving it as meaningful and purposeful, enjoying positive relationships at work, and feeling committed to their jobs (Kun & Gadanecz, 2022).
*Happiness at work*	Fitriana et al. (2021) defined *happiness at work* as feeling good about one’s work, amd job characteristics, and feeling aligned with the organisation, reflected in pleasant judgements and experiences such as positive feelings, flow at work, moods, and emotions.
*Job satisfaction*	Judge et al. (2017) defined *job satisfaction* as the overall evaluative judgement one holds about one’s job. They further distinguished between overall satisfaction (one’s evaluation of the job as a whole) and facet satisfactions (which concern specific aspects of the job, such as work tasks, pay, promotions, or supervision).
*Workplace flourishing*	According to Rautenbach and Rothmann (2017) *workplace flourishing* comprises three dimensions: emotional, psychological, and social wellbeing.
*Flourishing at work*	*Flourishing at work* refers to a high state of *employee wellbeing* that arises from positive work experiences and the effective management of job-related factors (Redelinghuys et al., 2019).
*Thriving at work*	Spreitzer et al. (2005) conceptualised *thriving at work* as a psychological state characterised by employees’ vitality and learning.

**Table 2:** Glossary of terms, their definitions, meaning, and relevance to the work domain.

Concepts	Definitions	Interpretation	Application in the Work Domain
**Context-free wellbeing**	A continuum that reflects the relative attainment of an overall, personal subjective state of quality (Lomas et al., 2023).	This is an overarching, high-level concept. It integrates several life domains to provide an overall evaluation of a person’s wellbeing.	It is applied in work-related research when researchers wish to assess a person’s overall wellbeing.
Wellbeing domains *	These are life areas in which wellbeing can be experienced and evaluated, such as physical, occupational, social, financial, or spiritual wellbeing.	Wellbeing can be evaluated both across domains to yield an overall wellbeing assessment, or within specific life domains. Pincus (2024) observed that contemporary accounts identify between 4 and 17 domains with most frameworks including physical, social, and mental dimensions.	The work/er wellbeing literature distinguishes several work-related domains, including occupational, employment, career, and work wellbeing.
**Components of wellbeing**	These are elements that constitute wellbeing.	Across much of the Positive Work and Organisations literature, wellbeing is conceptualised as comprising two facets: Feeling good (the hedonic, or happiness aspect of wellbeing) Functioning well (the eudaimonic component of wellbeing). The hedonic facet is further divided into: Emotional wellbeing (an individual’s affective experiences) Evaluative wellbeing (life or domain satisfaction). These are high-level frameworks that define the broad facets of wellbeing. They can be applied to the overall wellbeing concept as well as to each of its domains.	These components are applied widely in the work/er wellbeing literature, examining aspects such as happiness at work and job satisfaction.
Models of wellbeing **	These are theoretically or empirically grounded structured accounts that elaborate the broad components of wellbeing described above into finer elements.	These models articulate specific facets of the hedonic and eudaimonic components of wellbeing, offering a coherent framework in which the components complement one another and interact. They often inform the development of measures and the design of interventions.	The PERMA+4 work-wellbeing model (Donaldson et al., 2022) is an example of such a model.
Predictors of wellbeing **	These are factors that are thought to shape wellbeing.	They are often grouped into demographic characteristics, internal influences (such as personality or attitudes), and external influences (such as living conditions or broader social contexts). Some are embedded in models of wellbeing.	Numerous models attempt to capture the predictors of work wellbeing. Examples include job design (Grant et al., 2011), person-environment fit (Kristof-Brown et al., 2005), and the job demand-resources (Bakker & Demerouti, 2007).
Population segmentations ***	These are terms that refer to the overall wellbeing of specific groups.	Wellbeing is frequently conceptualised and measured in different ways across groups (for example: child wellbeing, employee wellbeing, immigrant wellbeing). Such segmentation matters since the expression and aspects of wellbeing can vary across populations.	Within the work domain, wellbeing may be assessed in relation to specific populations (worker wellbeing, employee wellbeing, entrepreneur wellbeing, etc.). Across these populations, overall wellbeing is often operationalised as an aggregate of wellbeing across multiple life domains (psychological, physical, social, financial).
Levels of wellbeing ****	These are terms that describe high, low or other distinct wellbeing gradations.	Terms such as flourishing, languishing, suffering, or illbeing refer to particular levels of wellbeing. They can be applied to overall wellbeing and to wellbeing within specific life domains.	These terms are often applied to the work domain (for example: flourishing at work).

* The conceptual map does not depict all of the life domains mentioned by researchers. ** Models and predictors of wellbeing are not included in the conceptual map. *** The conceptual map includes only a small selection of the many work-related populations that researchers examine. **** The conceptual map includes only a small selection of the many terms used to denote different gradations of wellbeing.

**Table 3:** Overall and work-related wellbeing concepts.

Concept	Definition	Notes	Synonymous Terms
*Overall wellbeing*	A continuum that reflects the relative attainment of a personal subjective state of quality across key life domains (Lomas et al., 2023).	Represents “a life well lived.” Aggregates several wellbeing domains (such as physical, psychological, occupational, financial, and social wellbeing).	*General wellbeing, global wellbeing, complete wellbeing*
**Population segmentation**			
*Worker wellbeing*	A continuum that reflects a worker’s relative attainment of a personal subjective state of quality across key life domains.	The overall wellbeing of working people (Wijngaards et al., 2022). Similar to overall wellbeing, it aggregates several wellbeing domains.	
*Employee wellbeing*	A continuum that reflects an employee’s relative attainment of a personal subjective state of quality across key life domains.	The overall wellbeing of those employed by organisations (Wijngaards et al., 2022). Similar to overall wellbeing, it aggregates several wellbeing domains	
**Work-related domains**			
*Occupational wellbeing*	A continuum that reflects the relative attainment of a personal subjective state of quality in the occupational domain.	A concept that encompasses a person’s employment wellbeing, career wellbeing, and work wellbeing.	
*Employment wellbeing*	A continuum that reflects the relative attainment of a personal subjective state of quality in the employment domain.	Pertains specifically to the domain of employment, encompassing wellbeing in relation to one’s current employment circumstances, including unemployment, employment, overemployment, underemployment, and other related conditions.	
*Career wellbeing*	A continuum that reflects the relative attainment of a personal subjective state of quality in the career domain.	Refers specifically to the career domain, encompassing an individual’s longer-term career trajectory and mobility across workplaces and roles.	
*Work wellbeing*	A continuum that reflects the relative attainment of a personal subjective state of quality in their current work domain.	Denotes wellbeing specifically in relation to an individual’s present work situation.	*Workplace wellbeing, wellbeing at work*

**Table 4:** Definitions for overall work-related wellbeing components.

Concept	Definition	Notes	Synonymous Terms
*Overall happiness*	A continuum that reflects a person’s contentment and emotional experience across key life domains.	Includes both the emotional experience and the evaluative component. Aggregates several life domains (physical, psychological, occupational, financial, and social wellbeing).	*General/global/overall subjective wellbeing, general/global happiness*
*Life satisfaction*	A continuum that reflects a person’s overall contentment with life across key life domains.	Contains the evaluative component of happiness.	*Satisfaction with life.*
*Emotional wellbeing*	A continuum that reflects a person’s emotional experience across key life domains.	Contains the emotional experience of happiness.	*General/overall/global emotional wellbeing, hedonic/affective wellbeing*
**Population segmentation**			
*Worker happiness*	A continuum that reflects a worker’s contentment and emotional experience across key life domains.	Pertains to working people. Includes both an emotional experience and the evaluative component. Aggregates several life domains (physical, psychological, occupational, financial, and social wellbeing).	*Worker Subjective Wellbeing*
*Employee happiness*	A continuum that reflects an employee’s contentment and emotional experience across key life domains.	Pertains to employees. Includes both an emotional experience and the evaluative component. Aggregates several life domains (physical, psychological, occupational, financial, and social wellbeing).	*Employee Subjective Wellbeing*
*Worker life satisfaction*	A continuum that reflects a worker’s contentment with life across key life domains.	Pertains to working people. Contains the evaluative component of happiness.	*Worker satisfaction with life.*
*Employee life satisfaction*	A continuum that reflects an employee’s contentment with life across key life domains.	Pertains to employees. Contains the evaluative component of happiness.	*Employee satisfaction with life.*
*Worker emotional wellbeing*	A continuum that reflects a worker’s emotional experience across key life domains.	Pertains to working people. Contains the emotional component of happiness.	*Worker hedonic/affective wellbeing*
*Employee emotional wellbeing*	A continuum that reflects an employee’s emotional experience across key life domains.	Pertains to employees. Contains the emotional component of happiness.	*Employee hedonic/affective wellbeing*
**Work-related domains**			
*Happiness with one’s occupational state*	A continuum that reflects a person’s contentment and emotional experience within their current occupation.	Includes both the emotional experience and the evaluative component. Refers specifically to the occupational domain.	*Subjective occupational wellbeing*
*Happiness with one’s employment*	A continuum that reflects a person’s contentment and emotional experience within their current employment.	Includes both the emotional experience and the evaluative component. Refers specifically to the employment domain.	*Subjective employment wellbeing*
*Happiness with one’s career*	A continuum that reflects a person’s contentment and emotional experience within their career domain.	Includes both the emotional experience and the evaluative component. Refers specifically to the career domain.	*Subjective career wellbeing*
*Happiness with work*	A continuum that reflects a person’s contentment and emotional experience within their current work domain	Includes both the emotional experience of quality and the evaluative component. Refers specifically to the work domain.	*Workplace happiness, Happiness at work, subjective wellbeing at work, subjective work wellbeing*
*Satisfaction with one’s occupational state*	A continuum that reflects a person’s contentment with their current occupational state.	Includes only the evaluative component. Refers specifically to the occupational domain.	*Occupational satisfaction*
*Satisfaction with one’s employment*	A continuum that reflects a person’s contentment with their current employment.	Includes only the evaluative component. Refers specifically to the employment domain.	*Employment satisfaction*
*Satisfaction with one’s career*	A continuum that reflects a person’s contentment with their career.	Includes only the evaluative component. Refers specifically to the career domain.	*Career satisfaction*
*Satisfaction with work*	A continuum that reflects a person’s contentment with their current work.	Includes only the evaluative component. Refers specifically to the work domain.	*Job/work/workplace satisfaction*
*Occupation related hedonic wellbeing*	A continuum that reflects a person’s emotional experience within their current occupation.	Includes only the emotional component. Refers specifically to the occupational domain.	*Occupation related emotional/affective wellbeing*
*Employment related hedonic wellbeing*	A continuum that reflects a person’s emotional experience within their current employment.	Includes only the emotional component. Refers specifically to the employment domain.	*Employment related emotional/affective wellbeing*
*Career related* *Hedonic wellbeing*	A continuum that reflects a person’s emotional experience within their career.	Includes only the emotional component. Refers specifically to the career domain.	*Career related emotional/affective wellbeing*
*Work related hedonic wellbeing*	A continuum that reflects a person’s emotional experience within their current work.	Includes only the emotional component. Refers specifically to the work domain.	*Job/work/related emotional/affective wellbeing*
